# A Role for Programmed Cell Death in the Microbial Loop

**DOI:** 10.1371/journal.pone.0062595

**Published:** 2013-05-08

**Authors:** Mónica V. Orellana, Wyming L. Pang, Pierre M. Durand, Kenia Whitehead, Nitin S. Baliga

**Affiliations:** 1 Institute for Systems Biology, Seattle, Washington, United States of America; 2 Polar Science Center, Applied Physics Laboratory, University of Washington, Seattle, Washington, United States of America; 3 Genomatica, Inc., San Diego, California, United States of America; 4 Department of Molecular Medicine, University of the Witwatersrand and National Health Laboratory Service, Parktown, South Africa; 5 Department of Ecology and Evolutionary Biology, University of Arizona, Tucson, Arizona, United States of America; 6 Integral Consulting Inc., Seattle, Washington, United States of America; 7 Department of Microbiology, University of Washington, Seattle, Washington, United States of America; University of California, Merced, United States of America

## Abstract

The microbial loop is the conventional model by which nutrients and minerals are recycled in aquatic eco-systems. Biochemical pathways in different organisms become metabolically inter-connected such that nutrients are utilized, processed, released and re-utilized by others. The result is that unrelated individuals end up impacting each others' fitness directly through their metabolic activities. This study focused on the impact of programmed cell death (PCD) on a population's growth as well as its role in the exchange of carbon between two naturally co-occurring halophilic organisms. Flow cytometric, biochemical, ^14^C radioisotope tracing assays, and global transcriptomic analyses show that organic algal photosynthate released by *Dunalliela salina* cells undergoing PCD complements the nutritional needs of other non-PCD *D. salina* cells. This occurs *in vitro* in a carbon limited environment and enhances the growth of the population. In addition, a co-occurring heterotroph *Halobacterium salinarum* re-mineralizes the carbon providing elemental nutrients for the mixoheterotrophic chlorophyte. The significance of this is uncertain and the archaeon can also subsist entirely on the lysate of apoptotic algae. PCD is now well established in unicellular organisms; however its ecological relevance has been difficult to decipher. In this study we found that PCD in *D. salina* causes the release of organic nutrients such as glycerol, which can be used by others in the population as well as a co-occurring halophilic archaeon. *H. salinarum* also re-mineralizes the dissolved material promoting algal growth. PCD in *D. salina* was the mechanism for the flow of dissolved photosynthate between unrelated organisms. Ironically, programmed death plays a central role in an organism's own population growth and in the exchange of nutrients in the microbial loop.

## Introduction

The microbial loop model is fundamental to our understanding of biogeochemical cycling of nutrients and minerals in aquatic eco-systems [1], [2]. Metabolic processes are distinctly coupled in microbial communities: photosynthetic primary producers (bacteria and/or unicellular algae) release C- and N-based dissolved organic matter (DOM) comprising various organic compounds and amino acids that are readily assimilated and re-mineralized by heterotrophic bacteria/archaea and protozoa [3], [4]. Phytoplankton and bacteria are the main sources of DOM [5], [6], [7], [8], [9]. These biopolymers are produced by several mechanisms including direct release [10], mortality by viral lysis [11], [12], [13], [14], [15], regulated exocytosis of metabolites and polymergels [16], grazing [17], [18] and apoptosis [19]. Apoptosis, the commonest phenotype of programmed cell death (PCD), is well documented in chlorophytes [20], [21] allowing cellular materials to become dissolved in the environment.

Much is known about C and N cycling in the aquatic microbial loop; however, in hypersaline environments the interactions are largely unexplored. The physiological complexity of the microbial population in these environments offers the potential for a staggering number of interactions and biogeochemical interdependencies [22]. To address this gap in knowledge we focused on one such environment, the Great Salt Lake (GSL) in Utah, USA. Photosynthetic eukaryotes and halophilic archaea (hereon "haloarchaea” in the GSL metabolize vast amounts of C (145 gC m-^2^ year^−1^) [23]. Important players in this environment are *Dunalliela salina* a unicellular photosynthetic chlorophyte, highly adapted to large changes in salinity, pH and temperature and *Halobacterium salinarum*, a halophilic archaeon that also thrives in environments with a range of salinities (116.88–292.2 PSU, temperatures (30° C–50° C), light intensities, oxygen tension and nutrients [24]. For both organisms glycerol is a major component of the C cycle. In response to salinity stress, *D. salina* enhances CO_2_ assimilation and channels the carbon and energy resources towards synthesis of glycerol (reaching internal concentrations as high as 7 M [25]), which it uses as an osmoprotectant [22]. When stressed, up to 17% of this glycerol can be found extracellularly and concentrations can reach as high as 30 µM after the demise of *Dunaliella* blooms [26]. *H. salinarum* is also well adapted to hypersaline conditions [27], [28], [29], [30]. The archeon responds well to a range of environmental stresses [31], [32] including low oxygen tensions [33], [34], [35] and fluctuating nutrients [30], [36]. Due to their adaptations to these environments, *Halobacterium* spp. can reach high abundances. *H. salinarum* and *D. salina* co-habit and are the most important organisms involved in hypersaline biogeochemistry [24]. Little is known about their physiological interactions, which became the immediate focus of this study.

While examining the flow of C (using glycerol as a proxy of DOM [26] between *H. salinarum* and *D. salina* a second question emerged. It was observed that PCD occurred in *D. salina* and its role in the nutrient exchanges between the two organisms was examined further. Algal release of DOM by a process that results in cell death has been reported and discussed primarily as a response to control cell growth under stressful and nutrient limiting conditions [19], [37], [38], [39], [40], [41], [42] (although not under carbon limiting conditions as described in this manuscript). However, the implications of these arguments are seldom examined in any detail and may rightly be criticized as naïve group selectionist thinking [43]. There are many possible evolutionary explanations (both adaptive and non-adaptive) for PCD in the unicellular world [42]. The fitness effects of unicellular PCD have also been examined in at least two model organisms: *Chlamydomonas reinhardti* [44], a relative chlorophyte of *Dunalliela*, and the yeast *Saccharomyces cerevisiae* [37] and been considered in phytoplankton ecology [45]. Here, we examined the role of PCD on a chlorophyte's population growth and the exchange of nutrients between two co-occurring organisms.

Surprisingly, we found that the growth rate of a *D. salina* culture can increase even though a significant proportion of its own population 55%+/−15 undergoes PCD at night after daytime growth. The benefit of releasing photosynthetically fixed C by PCD is achieved by increased growth measured as C-assimilation due to a positive metabolic feedback loop from itself when in pure culture, as well as from a co-habiting heterotrophic archaeon. Furthermore, the heterotrophic haloarchae *Halobacterium salinarum* can subsist entirely on the lysate of apoptotic algae. These data indicate that not only can PCD in *D. salina* benefit others of its own kind, but carbon released in PCD materials is recycled between the chlorophyte population and co-occurring unrelated haloarchaea.

## Materials and Methods

### Ecological sampling

Water was sampled at the South (Lat: 40°43′N Long: 112°13′W) and North (Lat: 41°26′N Long: 112°40′W) Arms of the Great Salt Lake (GSL; UT, USA) with autoclaved bottles and transported to the laboratory on ice. These studies do not involve protected species and no specific permits were required for the described field studies in the public areas of the GSL. *Dunaliella* spp. and haloarchaeal cells were isolated by enrichment in Minimal Media (MM1) liquid media (*Dunaliella*) at salinities ranging from 36, 75, 100, 150, 200, and 250 PSU. MM2-agar containing plates at the same salinities as for *Dunaliella* were used for haloarchaeal cells (see Supplement below).

### Species identification by DNA Sequencing

To survey the diversity of organisms that naturally co-exist in hypersaline environments we enriched photoautotrophs from the South-Arm of the GSL by inoculating water samples into MM1 media containing only salts at different concentrations (75, 100, 150, 200, 250 PSU) (Fig. S1 A). Exponentially growing *Dunaliella spp*. cells were harvested by centrifugation (2300 rcf×4 min); DNA extraction and purification were done using DNeasy Plant Mini Kit (Qiagen), and quantified spectrophotometrically. Species-specific internal transcribed spacers (ITS-1, and ITS-2) of the RNA genes, were amplified using the primers described in [46]. Secondly, to identify co-existing microbial species, aliquots of GSL water were plated onto nutrient rich solid agar medium over a similar salinity range as for the autotrophs. Bright pink, red and orange colonies grew (Fig. S1 B) that have characteristic colony morphologies of haloarchaea due to their high cellular content of bacterioruberins, carotenoids, and rhodopsins. Isolated colonies of haloarchaeal cells (Fig. S1 B) were then grown to exponential phase and harvested by centrifugation. 16 s rRNA sequences were amplified from genomic DNA extracts according to the PCR protocol described by [47]. All amplicons were sequenced using an ABI 3730xl DNA Analyzer.

### Culture conditions

For single and co-culture experiments, axenic batch cultures of *Dunaliella salina* (Culture Collection of Algae and Protozoa, UK, CCAP 19/18) were grown in artificial sea water (ASW [48] containing salts reaching a total of 200 PSU and enriched with nutrients as in f/2 media [49] called MM1. Cultures were grown under a 13 h∶11 h light∶dark photoperiod and at 150 µmol photons m^−2^ s^−1^ (verified by a Li-Cor 191SA (Li-Cor Inc.) at 30°C and 100 rpm shaking. Cells were entrained to this light regimen for 3 tandem culture transfers until the growth rate did not change (3 weeks) prior to the experiments. Growth rates were determined by the change in cell number and/or change in red autofluorescence (680 nm) over time. *H. salinarum* NRC-1 was maintained in CM media [50] at a salinity of 200 PSU and for co-culture experiments cells were grown in MM1 enriched with nutrients akin to DOM, including a mixture of amino acids (see supplement Table S1, for details) and acclimated to the same light∶dark photoperiod as *D. salina*. The inoculation density of co-cultures was 10^5^ ml^−1^ *D. salina* cells, and 10^8^ ml^−1^ *H. salinarum* cells. All experiments were run in triplicate. *D. salina* cells were counted by flow cytometry, *H. salinarum* was measured as optical density at 600 nm (OD_600_ of 1.0 = 8×10^8^ cells/ml).

### Duirnally synchronized syntrophic interaction between *D. salina* and *H. salinarum*

To investigate the physiological exchange and interplay between *D. salina* and *H. salinarum*, supernatant from a light/dark adapted *D. salina* culture was collected and seeded with *H. salinarum*. Culture growth was monitored at 37°C for 7 days using a BioscreenC high throughput microbial growth analysis instrument. Sterile MM1, MM1 with 400 mM glycerol and MM1 supplemented with amino acids (60 µM; see supplement Table S1 for a list of amino acids) commonly found in hypersaline ecosystems were used as controls.

### Single cell analysis and fluorescent cell stains

An Influx flow cytometer (Cytopeia) using a Coherent Innova 305C argon ion laser excitation source tuned at 488 nm and 200 mW was used to quantify cell abundance. Cell count was based on forward angle light scatter (FALS) and red fluorescence collected with a 610 nm long pass and a 700/50 nm band pass filter. Sampling was done every two hours with a calibrated robot. The cells were fixed in paraformaldehyde (0.1%) and kept at 4°C until counting. Yellow/green fluorescent 1μm microspheres (Polysciences, Warrington, PA, USA) were used to calibrate gain and object detection threshold settings and as an internal fluorescence standard for normalization and counting.

Intracellular glycerol-containing vesicles in *D. salina* cells were observed according to standard protocols by staining with the highly specific aminoacridine dye 1 µM Quinacrine [16], [51], [52] for 15 minutes and washed twice in 200 PSU saline. Cells were analyzed for green fluorescence of quinacrine (ex: 488 nm, em: 504–523 nm, 1 µM) using a 500 nm long pass (LP) and a 530/30 nn band pass (BP) filter and red autofluorescence of chlorophyll using a 610 nm LP and a 715/50 nm BP filters. Loss of quinacrine fluorescence from pre-labeled cells indicates vesicle secretion.

DNA in live cells was detected by staining with SYBR I (λex = 497 nm/λ_em_ = 520 nm, 3 µM Invitrogen) [53] or DAPI (4',6-diamidino-2-phenylindole, dihydrochloride) (ex: 350 nm em: 450 nm, 3 µM) and analyzed with a BD FACS ARIAII flow sorter and a Delta Vision confocal microscope. SYTOX® blue (ex: 440 nm, em: 525 nm, 3 µM), an impermeable probe, was used to detect dead cells with compromised plasma membranes. It was used in combination with the Annexin V stain for PCD detection (see below). *Dunaliella* sub-populations were defined by a combination of their forward scattering characteristics and their red autofluorescence of chlorophyll *a*. Populations were also identified by their red autofluorescence of chlorophyll *a* in combination with the fluorescence of the labeled probes used (FITC-Annexin, SYTOX® blue) and subsequently sorted. After sorting with the BD FACS ARIAII, the cell's membrane and nucleus integrity were evaluated and photographed with a Delta Vision confocal microscope. Data analysis was done using FlowJo analytical software (Tree Star).

### Programmed cell death (PCD) analyses

Three independent markers of PCD in chlorophytes were used [54], [55]: phosphatidylserine (PS) externalization, caspase activity and observation of the typical morphological changes associated with PCD in algae. Caspase activation and morphology are qualitative assessments; PS externalization allows qualitative and quantitative (cell enumeration) evaluation. The TUNEL assay (detection of DNA fragmentation flow cytometrically) was avoided for cell quantitation as it may detect non-specific double stranded DNA breaks, which occur during necrosis [56] and its specificity has been questioned by [45].

- PS externalization was detected by fluorescein isothiocyanate (FITC)-labeled Annexin V adapted to the manufacturer's suggested protocol for *Dunaliella* (BD Pharmingen). Annexin is a a Ca^2+^-dependent phospholipid-binding protein that binds with high affinity for PS. PS is located on the cytoplasmic surface of the cell membrane, except in PCD when PS migrates to the outer surface of the plasma membrane. FITC-Annexin was used in combination with SYTOX® blue (SB), an impermeable probe that only detects dead cells when the plasma membrane is disrupted. Experiments were done in triplicate. Cells were photographed in a Delta Vision microscope.
- Cysteine protease activity of caspase-3, which is specifically associated with PCD in *Dunalliela* spp [54], [55], was measured using a Caspase-3 Fluorometric Assay Kit (Assay Designs Catalog No 907-014) according to the manufacturer's instructions. Caspase-3 exists as a proenzyme, becoming activated during the PCD pathway. This assay measures the conversion of a non-fluorogenic peptide Ac-DEVD-AMC substrate for caspase-3 to a fluorogenic product that emits light at 400 nm when excited at 360 nm. Caspase-3 activity was calibrated with a solution of 7-Amino-4-methyl coumarin at 5 µM in reaction buffer at 30°C and expressed as units of fluorescence relative to the equivalent fluorescence obtained from 5.56 units of fully active Caspase-3 when reacting with the Caspase-3 substrate according to the assay kit.
- Cellular morphological changes associated with PCD including cell shrinkage, vacuolization, plasma membrane blebbing, nuclear condensation and ejection of the nucleus were examined using a Delta Vision confocal microscope. Microscopic detection of PS externalization was also performed.

### Radioisotope tracing

C-flux was determined with radioisotope tracing by addition of NaH^14^CO_3_ to an activity of 1µCi^14^C ml^−1^ to light∶dark acclimated pure and co-cultures of *D. salina* and *H. salinarum* at an inoculating ratio of 10^5^ *D. salina cells*: 10^8^ *H. salinarum cells*. Total activity was determined using phenylethylamine that stabilizes radioactive counts [57]. *D. salina* and *H. salinarum* cells were collected by 2 µm and 0.22 µm filtration, respectively, and washed in 200 g/L saline. ^14^C uptake was halted with 250 µL 6 M HCl, incubated at room temperature (RT) for 30 min and prepared for counting with the addition of Ecosint (National Diagnostics). Samples were counted (disintegrations per minute, DPM) using a Tri Carb 2810 TR (Perkin Elmer) scintillation counter and carbon incorporation calculated according to [58] with modifications.

### *D. salina* glycerol production and release

Glycerol, an important constituent of DOM in hypersaline environments [26], was measured as follows. For bulk glycerol release experiments, 2 ml samples from pure *D. salina* and *D. salina* plus *H. salinarum* co-cultures were collected in 14 mL falcon tubes at each time point. A 500 µL aliquot was removed and fixed in paraformaldehyde to a final concentration of 0.1% for cell counting via a hemocytometer and flow cytometry. One-mL of the remaining sample was centrifuged (3500 rcf) for 15 min and the supernatant removed for extracellular glycerol measurements. The *D. salina* cell pellet was resuspended in 500 µL ddH_2_O to lyze cells and release intracellular glycerol. Glycerol measurements were done using a free glycerol detection kit (Sigma F6428) as per the manufacturer's instructions.

### Transcriptional response of *H. salinarum* to *D. salina* dissolved organic matter (DOM)

Microarrays were generated at the Institute for Systems Biology Microarray Facility and processed according to standard protocols [59]; [60]. Each microarray slide contained 70mer oligonucleotides for each of the 2400 genes of *Halobacterium salinarum* [61] spotted in quadruplicate at two spatially distinct locations. Labeling, hybridization and washing were performed as described previously [31]. Statistical significance of differential gene expression was determined using the maximum likelihood method [62]. Data reported in this paper has been deposited in the Gene Expression Omnibus (GEO) database record GSE45752 (http://www.ncbi.nlm.nih.gov/geo/query/acc.cgi?acc=GSE45752).

#### Carbonate system

Total CO_2_ and Alkalinity were calculated according to Dickson et al. [63] (http://andrew.ucsd.edu/co2qc/index.html), pH was measured spectrophotometrically [64], and the salinity and temperature of the cultures (200 PSU, 30°C), and ambient pCO_2_ measurements (pCO_2_ = 400) were used for calculations.

#### Nutrients

phosphate, nitrate, nitrite, and ammonia were measured at the University of Washington Marine Chemistry Laboratory (http://oceanweb.ocean.washington.edu/services/techservices.html).

### Growth model

A growth model for both pure and co-cultures of *D. salina* and *D. salina + H. salinarum* was developed using the technical computing environment MATLAB (Mathworks, Inc.). The model was defined as a single birth-death ODE for the change in total cell density,
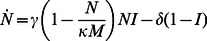
(1)with light irradiance,
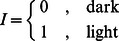
(2)a “memory” variable,
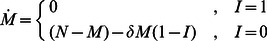
(3)and parameters γ, δ, and κ representing the maximum cell growth rate, cell death rate, and a saturation scaling factor, respectively.

Cellular growth, defined by the first term of Eq. (1), only allows N to increase during the light phase (I = 1) with a saturation level defined by *κM* (a Droop growth model) [65]. Importantly, Eq. (3) is such that 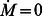 for light phases, providing a constant saturation threshold therein. Conversely, M resets to the value of N during dark phases. Thus, M allows the saturation level to increase with each diurnal cycle, a characteristic observed in experimental data. Cell death, defined by the second term of Eq. (1), only occurs during the dark phase (I = 0), and is modeled as simple exponential decay based on the assumption that the cells have little to no tolerance to darkness.

Light irradiance (I, Eqn. (2)) was simulated as a square wave over a range of 0 (dark) and 1 (light) with a duty cycle equivalent to the nominal experimental photocycle of 13 hr∶11 hr light∶dark (45.8%).

The model was fit to experimental data by iteratively adjusting the values of γ, δ and κ until the sum of squared errors between the predicted trajectory for N (numerical solution of Eqns. (1)–(3)) and experimental data was minimized. Model fits were done individually on each experimental replicate within each culture set to generate statistics for each parameter.

## Results

### Microbial organisms (chlorophytes and bacteria) in the Great Salt Lake

**Figure 1:**
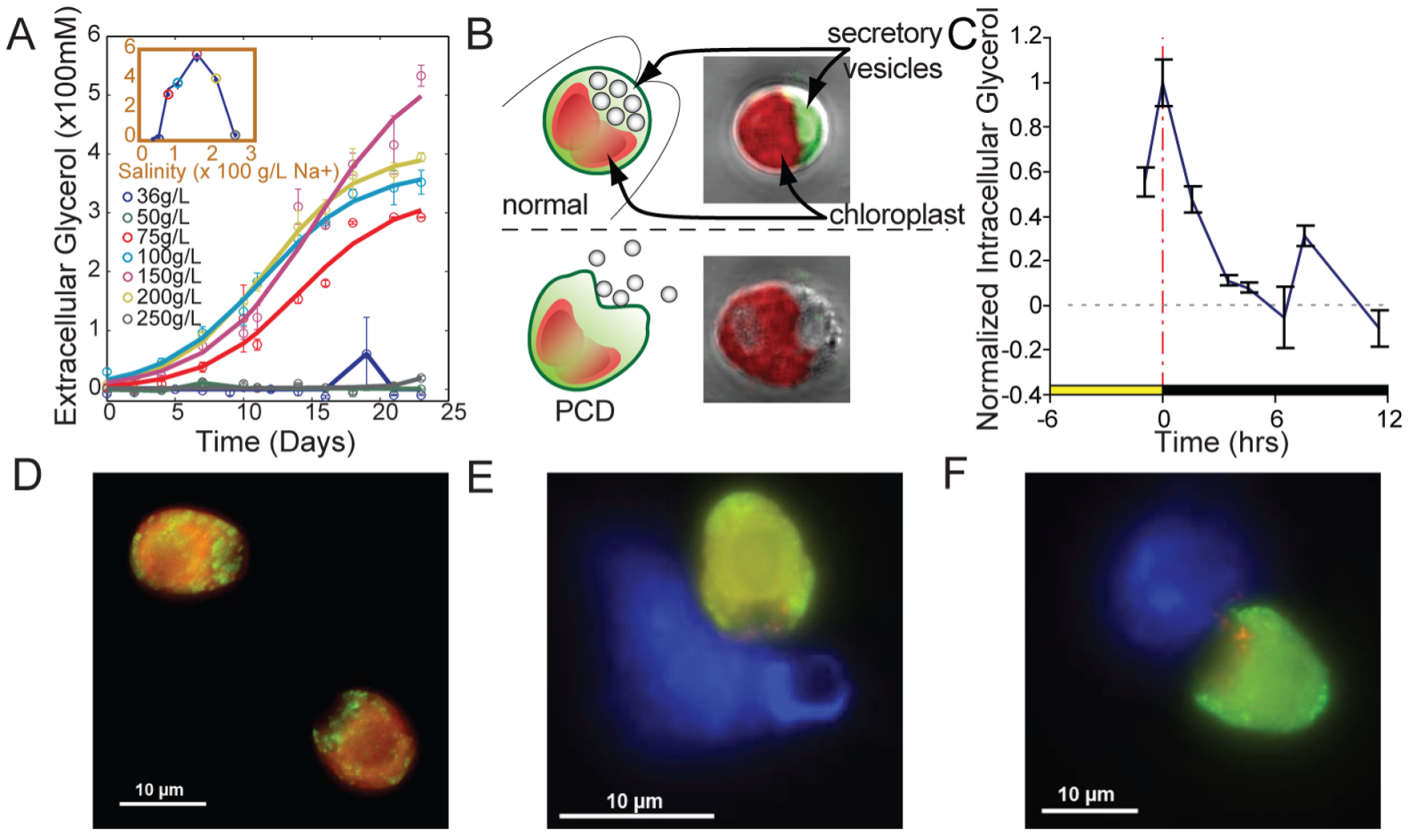
Cell death upon exposure to darkness or *H.salinarum* cells triggers the release of glycerol by *D. salina*. (A) *D. salina* accumulates and utilizes glycerol as an osmoprotectant in hypersaline growth conditions. Accumulation of glycerol in *D. salina* cultures is correlated to increasing salinity in the growth medium peaking at 150 PSU (inset). (B) *D. salina* releases glycerol by cell death. Illustrated and merged phase contrast/fluorescence photomicrographs of a *D. salina* cell undergoing cell death. *D. salina* stores glycerol and other byproducts of photosynthesis inside secretory vesicles that are localized to the apical flagellar pole (top). The green color of the vesicles is due to quinacrine staining of glycerol and the red fluorescence corresponds to chloroplasts. The image show dramatic disruption of the cell membrane and complete loss of internal glycerol in a cell that has undergone cell death (bottom). (C) A shift of live light acclimated cultures (100–150 µmol-photon m^−2^sec^−1^) to complete darkness (0 µmol-photon m^−2^ sec^−1^) results in release of glycerol by *D. salina*. The intracellular glycerol is measured by flow cytometry analysis of quinacrine stained vesicles *D. salina* cells. (D) Representative confocal laser micrograph of *D. salina* cells stained with FITC-Annexin V to highlight the externalization of PS (green fluorescence), and red corresponds to red chlorophyll autofluorescence. (E) Representative confocal laser micrographs of dead *D. salina* stained with FITC-Annexin V and SYTOX® blue highlighting PS completely externalized and the ejection of the nucleus indicating cell death.

The enriched algal population (**Fig. S1A**)was identified as predominantly belonging to *Dunaliella spp*, single-celled wall-less photosynthetic chlorophytes, specifically *D. salina, D. pseudosalina, D. viridis, D. parva*, and a few other species (**Fig. 1B** **; Fig. S2, A & B**). The microbial populations were all confirmed as halobacteria, with several direct matches to *H. salinarum* (**Fig. S2 C**), a halophilic photoheterotrophic archaeon that thrives over a wide range of salinity (135–300 PSU). These results confirm that *Dunaliella spp* co-exist with diverse halobacteria in the South Arm of the GSL [24]. *Dunaliella spp* density fluctuated over several months between 10^3^–10^8^/L and the density of haloarchaea fluctuated between 10^6^ and 10^9^/ml. Thus we choose *D. salina* and *H. salinarum* as a model system to study their interaction in laboratory experiments.

### Mechanism of DOM production by *D. salina*

The mechanism for release of photosynthetically sequestered C by *D. salina*, the predominant primary producer in halophilic environments [24], [26], was investigated. In response to routine increases in salinity, *D. salina* enhances CO_2_ assimilation to produce and store glycerol for use as an osmoprotectant [66]. This was corroborated here and glycerol production by *D. salina* peaked at its preferred salinity range between 150–200 PSU (**Fig. 1A**). Microscopy and quinicrine fluorescence revealed that glycerol stores are localized in vesicles (**Fig. 1B**) in *D. salina*. It is known that moieties for export are stored in vesicles [16], [67] and in natural settings, up to 17% of this glycerol reserve is released along with other uncharacterized metabolites into the environment [26].

To characterize the timing of glycerol release as a proxy of DOM release with respect to the diurnal cycle, intracellular glycerol was measured during the transition from day to night. There was a precipitous drop in intracellular glycerol upon transfer of light acclimated(≥150 µmol photon m^−2^ s^−1^) culture to complete darkness (0 µmol photon m^−2^ s^−1^) [40] (**Fig. 1** **B & C and Video S1**). A high percentage of cells releasing glycerol and other uncharacterized C and N metabolites died during this process and exhibited membrane damage. The percentage of FITC-annexin V in combination with SYTOX® blue labeled cells (**Fig. 1** **D & E**) detected the externalization of PS, confirming that 65+/−15% of cells were undergoing PCD. The proportion of apoptotic cells increased after the onset of darkness with a mean value of 59%. Previous observations also revealed that *D. salina* cells undergo PCD when placed in complete darkness and that this process is blocked by inhibitors of caspases, which are the effectors of apoptosis [40]. The cells stained with FITC-Annexin V showed the range of PCD-related morphological changes, from membrane blebbing to ejection of the cell nucleus followed by complete dissolution of the cells into the media (**Fig. 1** **E & F, and Video S1**). In addition, a one-to-one correlation between numbers of cells undergoing PCD and amount of extracellular glycerol was observed (see below). These findings indicate that glycerol and other uncharacterized PCD materials are released by active cell death in *D. salina* in hypersaline environments. The stimulus for PCD and subsequent glycerol and DOM release was the onset of darkness.

### Duirnally synchronized syntrophic interaction between *D. salina* and *H. salinarum*

**Figure 2:**
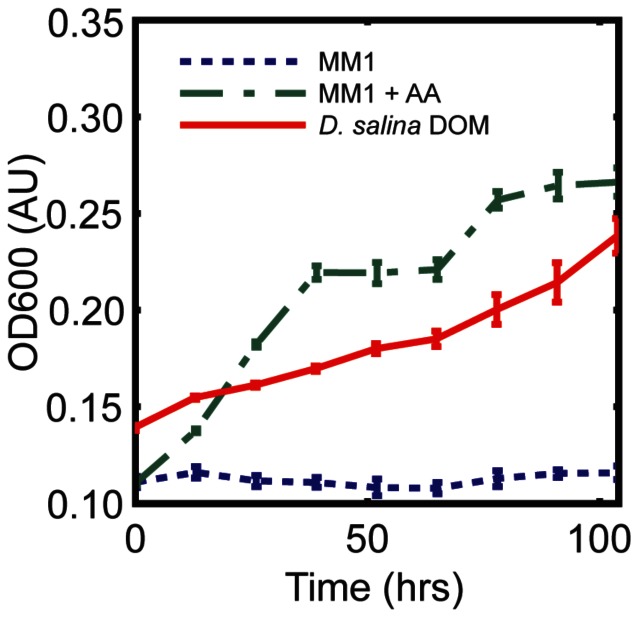
Dissolved organic material (DOM or photosynthate) released by *D.salina* fully complements nutritional requirements of *H. salinarum*. Supernatant of *D. salina* culture in artificial seawater (MM1) supported *H. salinarum* growth at a level that was comparable to its growth in MM1 supplemented with amino acids at naturally occurring concentrations.

Neither the MM1 media nor glycerol supplementation alone supported *H. salinarum* growth. The PCD supernatant from the *D. salina* culture containing DOM (glycerol, plus unknown C and N containing nutrients not measured), on the other hand, was nearly as supportive of haloarchael growth as MM1 supplemented with amino acids (Fig. 2). Thus, DOM released by diurnally synchronized cultures of *D. salina* fully complements nutritional requirements of *H. salinarum*.

**Figure 3:**
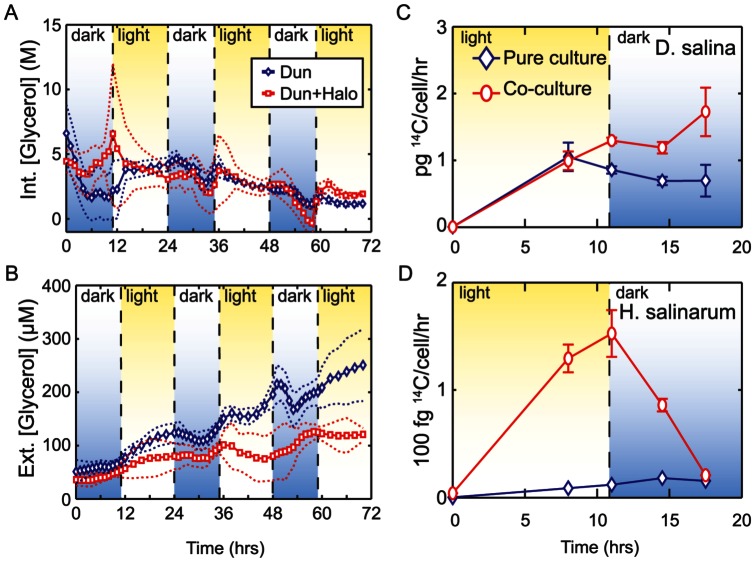
Diurnally synchronized syntrophic interaction with *H.salinarum* increases productivity of *D. salina*. (A) Intra- and (B) extra-cellular glycerol concentrations in *D. salina* culture individually (blue) or with *H. salinarum* (red) over several day: night cycles, dash lines represent +/− standard deviation. (C) Radiolabel incorporation and tracing shows daytime uptake and nighttime release of ^14^C by *D. salina*. Uptake of ^14^C by *D. salina* at night is enhanced two-fold in co-cultures relative to pure cultures indicating nighttime assimilation of ^14^C in presence of *H. salinarum*. (D) Simultaneous tracing of C within *H. salinarum* cells demonstrates uptake and processing of ^14^C in sync with the diurnal cycle.

Next, the hypothesis that *D. salina* benefits from its association with *H. salinarum* was explored. The intracellular glycerol concentration in both pure and co-cultures cultures, showed a slight drop in concentration during the night, and ranged between 5–2 M over the three day experiment (**Fig. 3A**). While the extracellular glycerol and possibly other nutrients in the DOM accumulated in both cultures over time, the rate of accumulation was significantly slower in co-cultures with *H. salinarum* relative to pure cultures of *D. salina* (**Fig. 3B**). This slower accumulation of glycerol and nutrients in co-cultures was partly explained by radioisotope incorporation and tracing, which revealed that a) *D. salina* incorporated its own released DOC (dissolved organic carbon) at night when in pure culture (**Fig. 3C**) and that b) *H. salinarum* incorporated ^14^C-labeled nutrients from the algal DOM and released metabolized products. However, *H. salinarum* did not incorporate NaH^14^CO_3_ by itself (**Fig. 3D**). Subsequently at night *D. salina* re-assimilated DOM, specifically dissolved carbon, in the presence of haloarchaea as shown by at least two-fold higher productivity (C/cell/h) relative to pure culture (**Fig. 3C**). This is consistent with the heterotrophic capabilities of *Dunaliella spp*. [68] and known abilities of other marine microalgae to nocturnally assimilate organic compounds suggesting that this might be a general behavior and not unique to hypersaline algae [69].

**Figure 4:**
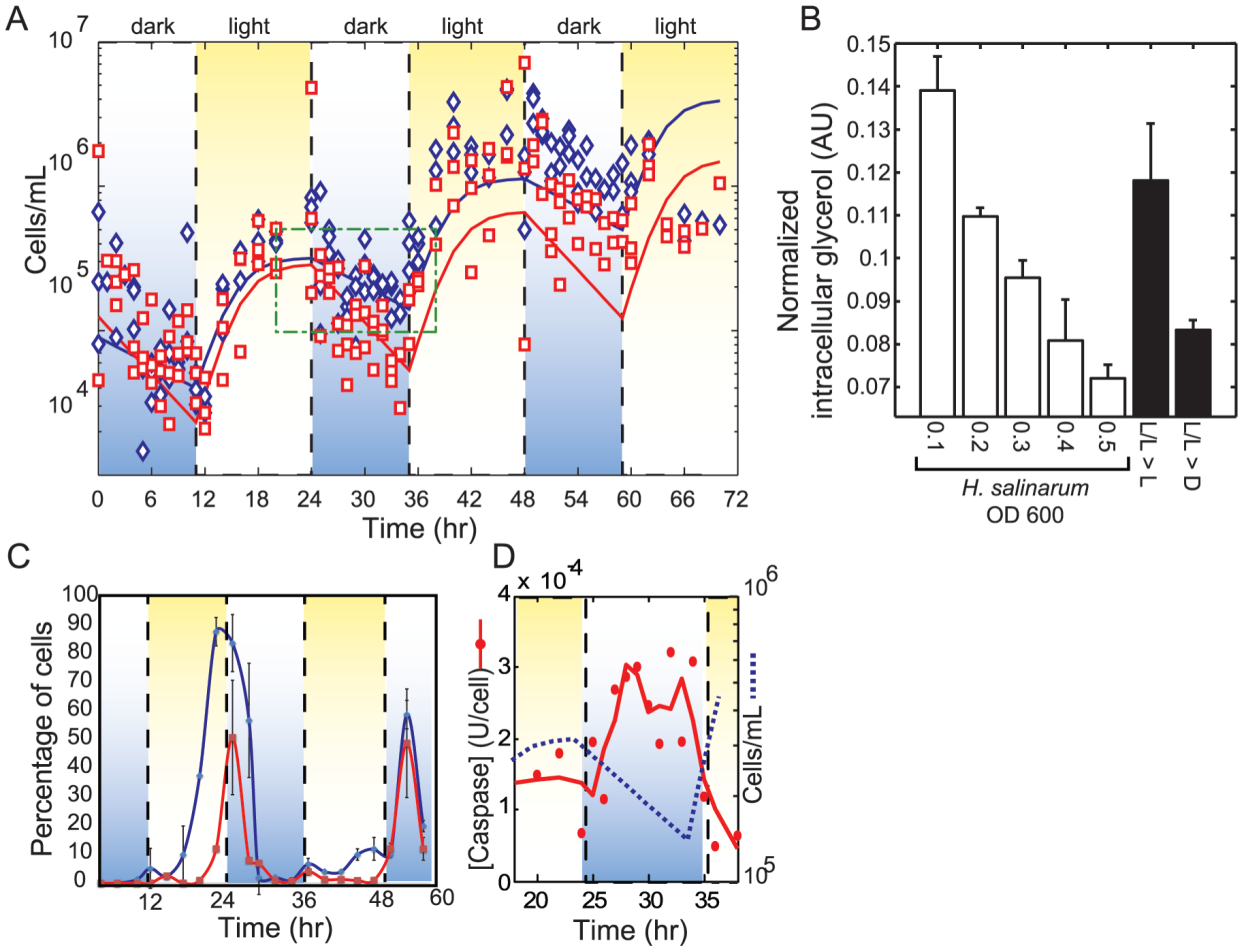
Cell death is triggered at nighttime as part of the diurnal synchronized program of *D.salina*. (A) Cell numbers for *D. salina* in pure and co-cultures with *H. salinarum* over several diurnal cycles. Live cell concentration measured using flow cytometry are indicated with blue (pure culture) and red (co-culture) points while lines are fitted model simulations. Green boxed region indicates time frame reported in **Fig. 3D** over which caspase-3 activity was assayed. (B) *H. salinarum* induces cell death in *D. salina* under continuous light regime. Intracellular glycerol within *D. salina* was stained with quinacrine and quantified with flow cytometry. Decrease of intracellular glycerol proportionally with higher cell density of *H. salinarum*. Unstimulated (pure *D. salina* culture and dark shifted samples are shown as controls. (L/L>L, cultures grown on a 24 h constant light regime maintained in the light during the measurements, LL>D, cultures grown in constant light shifted to dark conditions (0 µmoles m^2^s^−1^). (C) The decrease in *D. salina* cell number in the model (**Fig. 4A** (blue line) due to cell death is supported by the time course of annexin V labeled cells (blue line) indicating percentage of cells exhibiting externalization of PS and SYTOX® blue stained cells indicating the percent dead cells (red line). (D) The decrease in cell number in the model (blue dotted line) due to cell death is also supported by higher levels of caspase-3 during nighttime. Red line is Savitsky-Golay smoothed (span of 5) average of two replicate measurements for each time point.

To understand the implications of darkness induced cell death on algal population dynamics we constructed a growth model from experimental measurements of total cell counts (see methods) (**Fig. 4A**). The fitted parameters are shown in **Table 1**. The R-squared values (adjusted for degrees of freedom) for model fits to experimental data were 0.794+/−0.04 (s.e.m.) for pure and 0.702+/−0.09 (s.e.m.) for co-cultures. Our previous experimental data shows cell death during nighttime (**Fig. 4A & C**). Our model predicts that this occurs exponentially, and that the rate of decay is significantly faster (p<0.05) in co-culture (t_1/2_ = 5.9+/−1.4 hrs) than in pure culture (t_1/2_ = 10.1+/−2.8 hrs). Furthermore, the addition of increasing amounts of *H. salinarum* to *D. salina* cultures showed that *H. salinarum* independently induces algal cell death (**Fig. 4B** **and Video S2**). When daylight returns, surviving cells resume growth, saturating after a few divisions. Notably, the light induced burst growth rate is similar for both pure (t_doub_ = 1.1+/−0.6 hrs) and co-cultures (t_doub_ = 1.3+/−0.6 hrs). This process iterates over sequential day/night cycle resulting in daytime regeneration and nighttime drops in the algal population. Overall, both pure and co-cultures maintain a net positive growth rate, with the algal population doubling approximately every 13 hrs and 20 hrs in pure and co-culture, respectively, consistent with previously reported growth rates for *D. salina* [70].

**Table 1:** Initial and fitted parameter values for model of growth for pure *D. salina* and *D. salina* + *H*. *salinarum* co-cultures.

			Pure Culture			Co-Culture		
	Description	Units	Initial	Fitted	±CI_95%_	Initial	Fitted	±CI_95%_
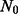	Initial cell density	Cells/mL	9000	–	–	12500	–	–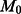	Initial cell density saturation threshold	Cells/mL	9000	–	–	12500	–	–
γ	Burst growth rate	hr^−1^	0.6262	0.678	0.406	0.4940	0.551	0.280
δ	Death rate	hr^−1^	0.0957	0.069	0.019	0.1141	0.119	0.027
κ	Saturation scaling factor	–	12.5813	8.10	2.73	12.5813	9.93	10.13

“±CI_95%_” are parameter 95% confidence intervals such that the lower and upper bound of estimated values are X-CI_95%_ and X+CI_95%_, respectively.

Furthermore, the *D. salina* cells externalized PS (a marker of PCD) a few hours before the actual death, the cells start dying just before the night period starts, with a peak immediately after the lights change into the dark period. These data indicate that death occurs at night, while cell numbers double during the day (**Fig. 4C**). This was also confirmed by measurements of caspase-3 activity, which were higher during times of cell death and low during the growth phase (**Fig. 4D**), and the morphological changes (**Fig. 1D & E**). Importantly, the intracellularly measured glycerol (∼4 M, assuming a volume of a secretory granule = 1 µm [71]) content in *D. salina* combined with the number of cells observed to undergo nocturnal cell death (∼3×10^5^ mL^−1^) accounted for the majority of the extracellularly measured glycerol (∼70 µM).

**Figure 5:**
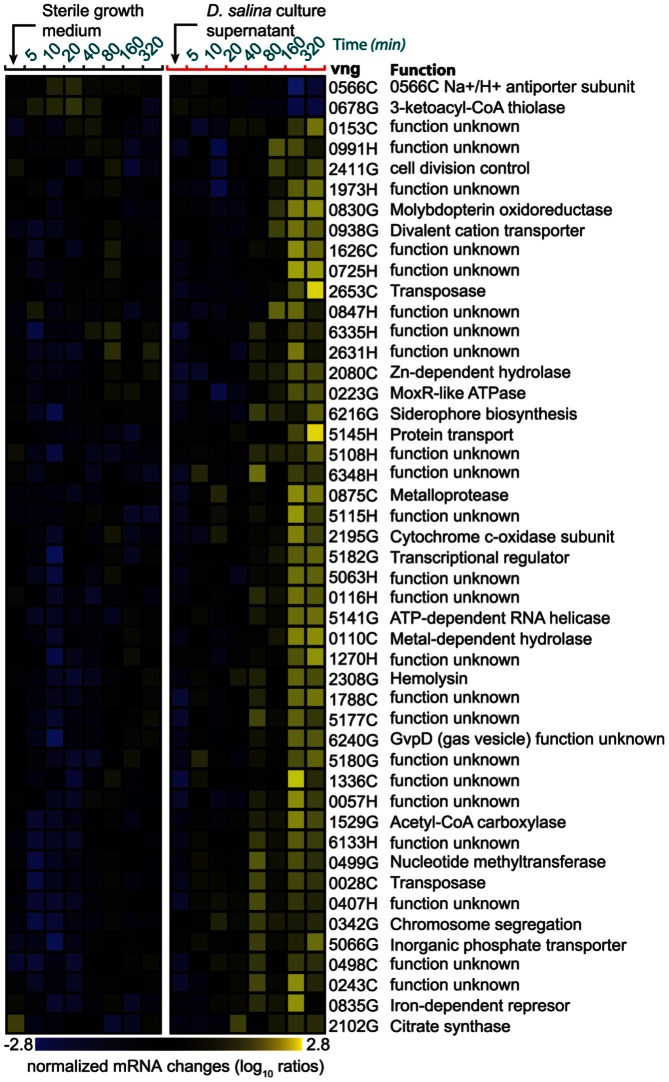
Mechanisms of communication and interactions in the syntrophic interaction. Transcriptional response of *H. salinarum* NRC-1 to *D. salina* conditioned artificial seawater amended with nutrients (MM1).

We also analyzed the transcriptional response of *H. salinarum* when challenged with the DOM released by *D. salina* (**Fig. 5**). At least 50 genes were differentially expressed and upregulated, of which nearly half are not annotated and are of unknown function [72], [73]. Those that had some putative functional assignment were mostly up regulated and included genes for siderophore biosynthesis, proteases, transport, metabolism, and cell division. Differential expression of a subset of these functional categories is consistent with known mechanisms for stimulation of algal productivity, nutrient uptake, and growth [74]. Similarly, up regulation of DMSO fermentation enzymes [75] and cytochrome oxidase suggests putative energy production mechanisms in haloarchaea engaged in this interaction, although DMSO production by *D. salina* is yet to be demonstrated. The upregulation of hemolysin a pore forming enzyme damaging to chlorophyte membranes may have accelerated *D. salina*'s cell death. Furthermore the induction of a putative protein transporter, hydrolases and several proteases might be important for the hydrolysis of macromolecular components in the algal photosynthate. Notably, proteases and other hydrolytic enzymes are known to be abundant in the marine and freshwater DOM [2], 76 In terms of the physical architecture of this interaction, we also observed that haloarchaeal cells were often physically associated with the algal cells both in co-cultures of laboratory strains and in the natural environment (**Fig. S3 A and B**), analogous to cellular interactions observed in the phycosphere [77], [78].

## Discussion

Interactions between members of different species play a fundamentally important role in all ecosystems and are driven by natural selection [79]. Interactions between bacteria and phytoplankton are well studied; bacteria exist as free living organisms in the phycosphere [78] attached to the surface of algal cells [77], intracellularly as algal symbionts [80] or attached to aggregates of cells and phytoplankton exopolymeric products [81] or microgels [82]. In contrast, archaeal-phytoplankton interactions, including their role in biogeochemical cycles, are poorly understood. Given the abundance of archaea on our planet (∼20% of total biomass on earth [83]), eukaryotic-archaeal syntrophic interactions and their metabolic mechanisms are central to understanding the cycling of C, N, and P in aquatic systems.

To explore this gap in knowledge, the cycling of C (in the form of glycerol) between *D. salina* and *H. salinarum* in a hypersaline environment, pure cultures and co-cultures of these species were used as a model. We found that darkness-induced PCD in *D. salina*, results in the active release of cell constituents such as glycerol and other unknown C and N, and P nutrients that can be used by others. At first glance, this seemingly altruistic behavior might appear perplexing. However, at high salinities (200 PSU) and high temperatures (30°C) CO_2_, the main source of carbon for photosynthesis, is limiting in the laboratory cultures and the GSL [84]. CO_2_ is very insoluble at high salinities (200 PSU) and high temperatures (30°C)[85], reaching a total concentration equal to 6.4 µM and an alkalinity equal to 76.8 µM in the experimental conditions (see methods [63]). This is similar to the titratable alkalinity in the GSL of 78 µM [23]. Furthermore, in the oceans even at lower salinities TCO_2_ is known to fluctuate from 10–15 µM, already posing a carbon limitation on phytoplankton cells [86]. Although *D. salina* possesses a carbon concentrating mechanism (CCM) [87], microbial mats from brine pools and hypersaline lakes have a diffusion-limited assimilatory pathway in which the isotopic fractionation by ribulose1,5- bisphosphate carboxylase is completely suppressed indicating CO_2_ limitation [85]. Furthermore CCMs can be modulated by temperature [88]. Thus, under the laboratory conditions designed to mimic the conditions experienced at GSL when other nutrients are present (NO_3_: 160 µM), carbon appears to play the role of a limiting nutrient [14], [19], [89], [90]. These data show that nighttime induction of cell death in a fraction of the algal population is a regulated process with no long term detriment to the net size of the algal population, on the contrary, PCD provides a carbon source to others that enhances population growth (**Fig. 4A**) in a carbon limited environment. This is an apparent evolutionary adaptation to live in such a hypersaline, carbon deplete environment.

**Figure 6:**
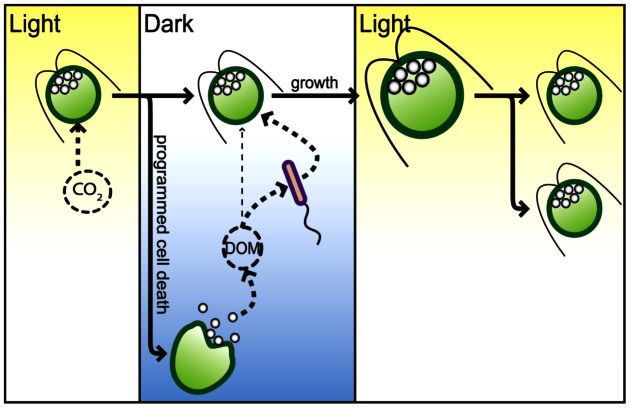
Diurnally synchronized cell death drives C-flux in an algal-archaeal syntrophic interaction. At night a stochastic process determines the fate of each algal cell resulting in up to 74% of cells undergoing death to release DOM (byproducts of photosynthetic C assimilation) into the surrounding media. The DOM are further metabolized and remineralized by archaea into a form that is readily consumed by algae. With onset of the subsequent day cycle, the algal population rapidly regenerates with up to 3 doublings with a cell division rate of 1.4 hrs. This entire process iterates over the next diurnal cycle.

Interestingly, *D. salina* photosynthate also complements the nutritional needs of *H. salinarum*, which independently induces algal cell death (**Fig. 4B** **and Video S2**). The archaeon is able to metabolize and remineralize DOM produced by PCD and carbon metabolites thereby providing elemental nutrients for the mixoheterotrophic chlorophyte (**Fig. 3C**). These data describe previously unknown physiological interactions in the hypersaline microbial loop and serendipitously, provided new molecular insights into the sociobiology of programmed cell death. Surprisingly, PCD in *D. salina* played an unexpected role in nutrient supplementation of its own population in a carbon limited environment (**Fig. 4C**). *H. salinarum* was able to exploit this process and use the DOM released by PCD to supplement its own population with carbon and nitrogen needs. Diurnally synchronized programmed death of *D. salina* was the mechanism for releasing sequestered C in the form of glycerol and other DOMs (amino acids and other unknown N and C and P containing compounds). This facilitated nutrient exchange with others in its own population to maintain positive growth (**Fig. 3C** **,** **Fig. 4A**) as well as a heterotrophic archaeon and possibly other organisms in the field not discovered in this study (**Fig. 6**). Our flow cytometry, biochemical and morphological data indicate that the death phenotype bears the hallmarks of apoptosis-like PCD [91]. In addition, detection of PS externalization permitted the quantification of PCD cells indicating that a high percentage of cells die via PCD before the onset of darkness suggesting an anticipation to photosynthesis decline and the cell's need for a heterotrophic carbon source (**Fig. 4C**). PS externalization is followed by other PCD markers like the loss of membrane integrity and nuclear condensation and eventually death as determined by SYTOX® blue staining (**Fig. 1** **D&E; Video S1**). Furthermore we also measured caspase 3 activity, which has previously shown to be associated with PCD in chlorophytes (**Fig. 4D**) [54], [55]. A mean of 57%+/−17% of the algal cells died each night and recovered at an equivalent rate with 2–3 cell divisions the subsequent morning (**Fig. 4A**). The equilibrium between nighttime population loss and daytime regeneration is a strong indicator that these are linked events.

The DOM released by algal programmed death complements the nutritional needs of co-inhabiting *H. salinarum*, which in turn (indirectly) supplements the requirements for further algal growth (**Figs 2** **&** **3C**). While the precise nutritional dependencies of *D. salina* and *H. salinarum* have yet to be characterized, algal dependency on bacteria and archaea generated metabolites such as vitamin B is well known [92], [93], as is the reciprocal archaeal and bacterial need for algal metabolites [94], [95]. It is significant in this regard that haloarchaea can further stimulate cell death in a density dependent manner (**Fig. 4 B**), to simultaneously regulate DOM production and acquire its nutritional necessities, controlling *D. salina* population dynamics by decelerating the algal population growth [96] (**Figs 4A & B**) and to control perhaps its own density suggesting a predator –prey dynamic maintaining ratio dependent densities which predicts proportional increases of both populations. While prey dependent models predict that only prey benefit from increase prey production [97]. In this case, *D. salina* population keeps on growing (**Fig. 4A**).

Thus, the increased rate of cell death in the co-cultures was perhaps due to an additive effect of cell death stimulation, by both changes in illumination (darkness induced PCD) and presence of haloarchaea producing hemolysin a pore-forming toxin to further lyse *D. salina* and increase the number of dead cells (**Fig. 5**). Although it was known previously that photoautotrophic algae and photoheterotropic haloarchaea [60] independently synchronize their physiologies with the light/dark cycle, our results demonstrate how such entrainment might enable their physiological cooperation. Furthermore, since bacteria/archaea can only take up molecules <650 Da [98], macromolecules can only be utilized if they are first hydrolysed outside the cell. This explains the upregulation of proteases and hydrolases by haloarchaea and the uptake of DOM during the day after it is released at night by *D. salina*.

A key evolutionary interest here is the role of PCD in this system. Programmed death in *D. salina* provides nutrients for its own population in a carbon limited environment. The haloarchaea exploit the process in a density dependent manner (**Fig. 4B**) and in turn (indirectly) supplements the requirements for further algal growth (**Fig. 3C**). Evolutionary theory predicts that such interactions can easily become unstable as “cheaters” (mutants that take advantage of “helpers”) take over the population. However, an alternate explanation comes from the Red Queen Hypothesis applied to populations and communities [99]. When natural selection acts at a level other than the individual organism interactions that increase the overall fitness of interacting species can achieve a dynamic equilibrium. Specifically, interaction with *H. salinarum* increases the overall fitness of *D. salina* as shown by improved primary productivity of the algal population after remineralization of DOM (**Fig. 3C**). Increased productivity generally results in increased growth rates, which taken together with the death of 57%+/−17% of its members might also help to maintain a young and healthy population of *D. salina*. In other words, while *H. salinarum* is supported by the photosynthetic activity of *D. salina*, it in turn becomes a helper to prevent the exhaustion of resources by improving algal productivity and, ultimately, the carrying capacity of the environment. Such behaviors are frequent when food is scarce and species are in competition [74], [100]. In hypersaline environments where carbon is highly insoluble and limiting, nutrients are rapidly exhausted after spring blooms [101]. However, if the chlorophyte-chlorophyte and chlorophyte-haloarchaeal interactions are stable year round [24], then such a strategy is evolutionarily advantageous for both species in this carbon limited environment. In these interactions we can then predict that the physical clumping of the algal and algal-haloarchaeal cells is likely to be critical for regulating this process and maintaining the structure and equilibrium of these interactions. Based on the fitness measurements (algal or haloarchaeal growth), PCD benefits both species. Since PCD is clearly not beneficial to the actor, this study supports the argument that the phenomenon is an altruistic adaptation at a level of organization other than the individual cell. Our data show that PCD may be beneficial for population growth in nutrient-limited environments; however there may be other reasons not explored here. For example cellular aging or population regulation as well as non-adaptive pleiotropy or epistasis may make as yet undiscovered contributions.

On a grander scale, by mediating such interplay with archaea and other organisms, diurnally synchronized cell death of algae is potentially an important determinant of microbial diversity and dynamics, succession of species, and biogeochmical cycles of C, N and P in natural aquatic ecosystems [95], [102]. As such, it also offers a new dimension to the structure of interspecies interactions in the microbial loop [1], [2] by adding to the diversity of mechanisms by which microbial interactions can occur. By characterizing the microbial loop in this manner we can now explore new strategies for predicting and managing the flux of C, N and other key elements of life with artificial (e.g. caspase inhibitors) or natural (e.g. archaeal mutants) modulators of algal cell death. Such strategies could also have interesting biotechnology applications to the controlled harvest of algal products. Ironically, programmed death takes center stage in the complexity of a living system that is the microbial loop.

## Supporting Information

Figure S1 **Enrichment of halophilic photoautorophs and heterotrophs from Great Salt Lake.** (A) Tubes containing Great Salt Lake (GSL) field samples enriched for photoautotrophs in MM1 with salinities from 36 to 250 g/L (left to right)–characteristically defined by their green pigmentation. (B) Rich medium agar plates in salinities from 36 to 250 g/L (left to right) used to enrich bacterial/archaeal species from GSL water samples.
(EPS)

Figure S2 **Halophilic biodiversity of the Great Salt Lake, Utah, USA.** Phylogenetic dendrograms of algal 18S rRNA regions ITS1 **(A)** and ITS2 **(B)** comparing GSL isolated photoautotrophs to *Dunaliella spp*. and other phytoplankton. Similarly, 16S rRNA genotyping of microbial heterotrophs isolated from the GSL **(C)** confirms the presence of *Halobacterium sp*. (an exact match for current lab strains) along with other halophilic archaea.
(EPS)

Figure S3 **Mechanisms of communication and interactions in the syntrophic interaction.** (A) Algal-bacterial/archaeal aggregates observed in (GSL) field samples enriched for photoautotrophs in 200 g/L salinity MM2. (B) *Dunaliella salina* and *Halobacterium salinarum* aggregates observed in 4-day old 13 hr∶11 hr light∶dark adapted cultures.
(EPS)

Table S1 **MM1 Composition (All concentrations in mM unless otherwise specified).**
(DOCX)

Video S1 **Timelapse merged brightfield and red/green fluorescence imaging of a representative** ***D. salina*** **cell undergoing exocytosis/cell death.** Red fluorescence is due to chlorophyll emission from 488 nm excitation. Green fluorescence corresponds to Quinacrine staining, which localizes to secretory vesicles at the flagellar pole of the cell.
(MOV)

Video S2 **Phase contrast timelapse imaging of** ***D. salina*** **cells undergoing exocytosis/cell death in response to** ***H. salinarum*** **NRC-1 cells.** To facilitate imaging and localized exposure to *H. salinarum* cells, *D. salina* cells were trapped within 10 µm deep microfluidic imaging chambers. The chamber at the top of the image receives *H. salinarum* injection, while the chamber at the bottom only receives sterile growth medium. *H. salinarum* cells are injected at the 10 min timepoint. Only a subset of the chamber population are observed to undergo exocytosis/cell death, consistent with total growth counts in diurnally adapted batch culture.
(MOV)
