# Supplementary material for: A Role for Programmed Cell Death in the Microbial Loop
Source: PLoS One. 2013 May 8;8(5):e62595. doi: 10.1371/journal.pone.0062595 (PMC3648572; doi:10.1371/journal.pone.0062595)
Supplement: Table S1 — MM1 Composition (All concentrations in mM unless otherwise specified). (DOCX) [file pone.0062595.s004.docx]

## Table S1

## MM1 Composition (All concentrations in mM unless otherwise specified).

### Salts

| Sodium chloride (NaCl) | 3.22 M |
| --- | --- |
| Magnesium sulfate heptahydrate (MgSO_4_*7H_2_O) | 40 |
| 3-morpholinopropane-1-sulfonic acid (MOPS) | 40 |
| Potassium chloride (KCl) | 27 |
| Sodium phosphate monobasic (NaH_2_PO_4_) | 167 µM |

### Vitamins

| Folic acid | 11.33 µM |
| --- | --- |
| Thiamine HCl | 14.82 µM |
| Biotin | 2.05 µM |

## MM1 Amino Acid Supplementation for H. salinarum growth

All concentrations in mM unless otherwise specified.

| L-alanine | 5.71 |
| --- | --- |
| L-arginine HCl | 1.9 |
| L-asparagine | 0.96 |
| L-aspartate | 1.88 |
| L-glutamate | 10.64 |
| L-glutamine | 5 |
| L-glycine | 1 |
| L-histidine HCl | 0.239 |
| L-isoleucine | 3.35 |
| L-leucine | 6.1 |
| L-lysine HCl | 1.04 |
| L-methionine | 0.603 |
| L-phenylalanine | 0.303 |
| L-proline | 0.4 |
| L-serine | 2.902 |
| L-theorine | 4.2 |
| L-tryptophan | 0.098 |
| L-tyrosine | 0.618 |
| L-valine | 2.135 |
| L-cysteine | 45 |
